# Persistent hyperparathyroidism secondary to ectopic parathyroid adenoma in lung: Case report

**DOI:** 10.3389/fendo.2022.988035

**Published:** 2022-12-13

**Authors:** Majid Valizadeh, Amir Ebadinejad, Atieh Amouzegar, Anahita Zakeri

**Affiliations:** ^1^ Obesity Research Center, Research Institute for Endocrine Sciences, Shahid Beheshti University of Medical Sciences, Tehran, Iran; ^2^ Endocrine Research Center, Research Institute for Endocrine Sciences, Shahid Beheshti University of Medical Sciences, Tehran, Iran; ^3^ Department of Internal Medicine, Emam Khomeini Hospital, Ardabil University of Medical Science, Ardabil, Iran

**Keywords:** ectopic parathyroid adenoma, hyperparathyroidism, video-assisted thoracoscopic surgery (VATS), embryologic anomaly, MIBI scan

## Abstract

Primary hyperparathyroidism (PHPT) is the most prevalent cause of hypercalcemia, affecting 0.3% of the population. The only curative procedure is parathyroidectomy. Persistent PHPT occurs in 4.7 percent of patients, even in the most skilled hands. Ectopic adenomas are challenging to localize before and during surgery and frequently result in persistent PHPT. We presented a case with persistent PHPT due to lung parathyroid adenoma that was successfully resected with video-assisted thoracoscopic surgery. A 55-year-old female patient was admitted to our endocrinology clinic with persistent PHPT after four neck explorations over 16 years. The last ^99^m Tc-MIBI scintigraphy with SPECT showed nothing suggestive of parathyroid adenoma, neither in the neck nor the mediastinum, but a solitary nodule as an incidental finding was reported in the lower lobe of the right lung, which was highly probable for a parathyroid adenoma in a fluorodeoxyglucose PET scan. Pathological examination ruled out parathyromatosis and lung malignancy; despite its location outside the anticipated embryonic pathway, pathology revealed the presence of an ectopic parathyroid adenoma. After the surgery, serum parathyroid hormone and calcium levels decreased, and hypoparathyroidism was corrected with calcium carbonate and calcitriol.

## Introduction

Primary hyperparathyroidism is defined by increased parathyroid hormone secretion resulting from parathyroid adenoma, hyperplasia, or, in rare cases, parathyroid malignancy (1). Parathyroidectomy is the treatment of choice in all cases of symptomatic PHPT (2). Effective surgery depends on the accurate identification and removal of parathyroid glands, which was historically accomplished by bilateral neck exploration due to a weakness in localization technique (3). Primary hyperparathyroidism that is persistent or recurrent is attributable to insufficient resection or the development of a second adenoma or malignancy (1); However, ectopic parathyroid glands continue to pose diagnostic and surgical challenges in patients with primary hyperparathyroidism (3). Difficulties in finding the ectopic parathyroid adenoma may cause a delay in localization and performing the surgery in which ectopic parathyroid glands can be located anywhere from the base of the tongue to the mediastinum (4). Here we present a 55-year-old woman who had several neck surgery over a 16-year period and was eventually diagnosed with an ectopic parathyroid adenoma in the lung. This case report has been reported in line with the CARE Criteria.

## Case presentation

**Figure 1:**
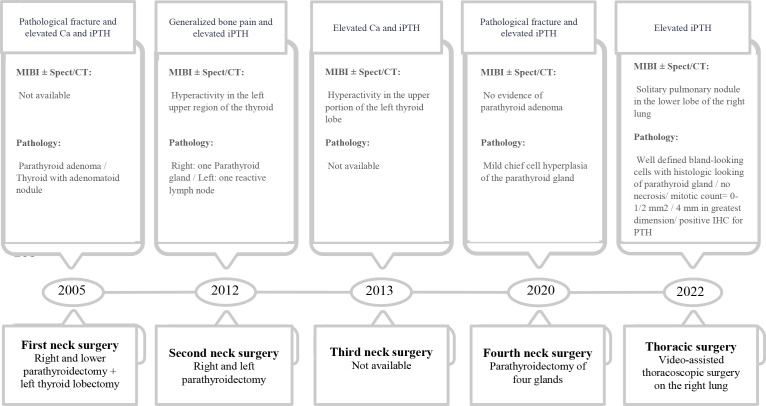
Details of patient surgeries during the course of the disease.

A 55-year-old woman with a history of chronic generalized pain and recurrent kidney stones was referred to our center in April 2021. In the last 16 years, the patient has undergone neck (exploration) surgery four times, however, PHPT has not been cured (**Figure 1**). She had a history of smoking for a long time. The patient’s previous medical history includes a hysterectomy at the age of 39 years following abnormal uterine bleeding, hypertension from 4 years ago, and several kidney stones. She has not any family history of endocrine diseases.

**Figure 2:**
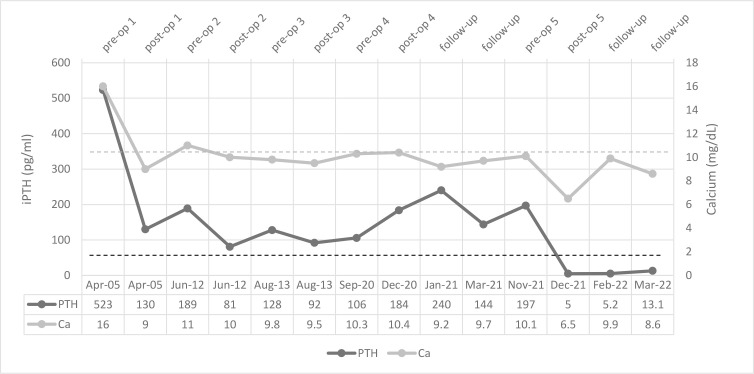
Serial serum levels of calcium and iPTH among clinical events. The dotted lines represent the reference lines.

She underwent orthopedic surgery sixteen years ago due to a pathological right tibia fracture that resulted in a nonunion. The patient had elevated levels of intact parathyroid hormone (iPTH) and serum calcium following surgery, despite the lack of additional diagnostic tests (**Figure 2**) The lower right parathyroid gland was excised, and the left thyroid lobectomy was performed without any early complications. Following surgery, serum calcium levels were corrected, while iPTH levels declined dramatically without being in the normal range (**Figure 1**). Pathological analysis of the parathyroid gland specimens identified the presence of a parathyroid adenoma.

The patient had been symptom-free for seven years prior to presenting with generalized body pain. She underwent a second neck exploration due to elevated serum iPTH and calcium levels and increased uptake in the left upper side of the neck on the 99mTc Sestamibi (MIBI) scan (**Figure 1**). Pathology showed one normal parathyroid gland on the right side and one reactive lymph node on the left (Ca = 11 mg/dl, iPTH = 189 pg/ml). Subsequent parathyroid surgery was performed a year later, following impaired iPTH and calcium and hyperfunction parathyroid tissue in the left upper portion of the thyroid lobe in the MIBI scan accompanied by findings of parathyroid adenoma in single-photon emission computed tomography/CT (SPECT/CT) scan (**Figure 1**).

She had a fracture in her left wrist seven years later with a poor healing process; the serum calcium level was up to 10.2 mg/dL and iPTH 106 pg/mL, 24-hour urinary calcium level was 214 mg. A SPECT-CT scan showed neither an ectopic mediastinal parathyroid adenoma nor any neck lesion. She underwent additional neck surgery due to high serum iPTH levels, while serum calcium levels remained in high normal reference ranges or slightly higher on two occasions. In the prior surgery report, the surgeon claimed that all four parathyroid glands were removed; however, pathology indicated just one hyperplastic parathyroid gland, and all excised tissue was reported to be nonspecific. Intact PTH levels remained high in all subsequent postoperative visits (**Figure 2**). Also, a dual-energy X-ray absorptiometry scan was done, and T scores of -4.0 at the lumbar spine and -2.7 at the femoral neck revealed osteoporosis.

**Figure 3:**
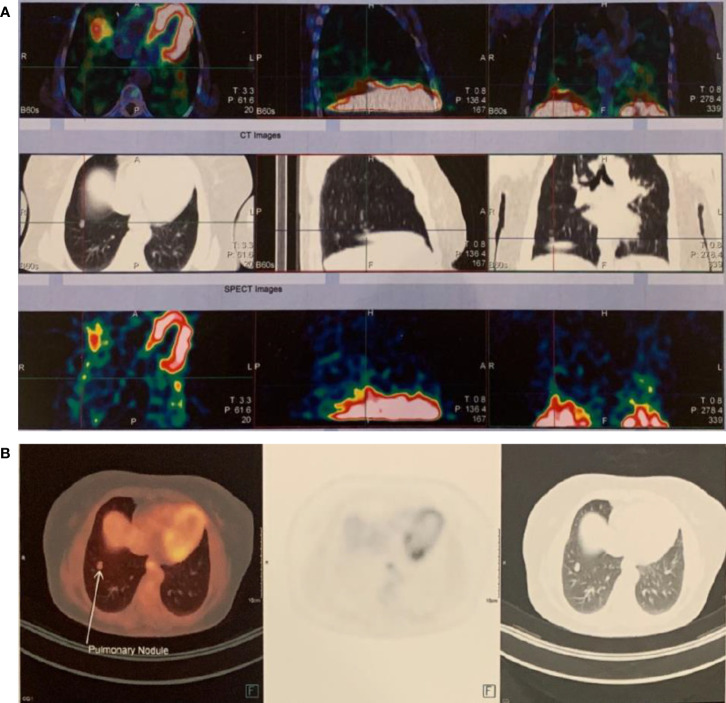
The ectopic parathyroid adenoma in the right lower lobe was demonstrated in **(A)** ^99^mTc Sestamibi scan, **(B)** 18F-FDG PET scan.

**Figure 4:**
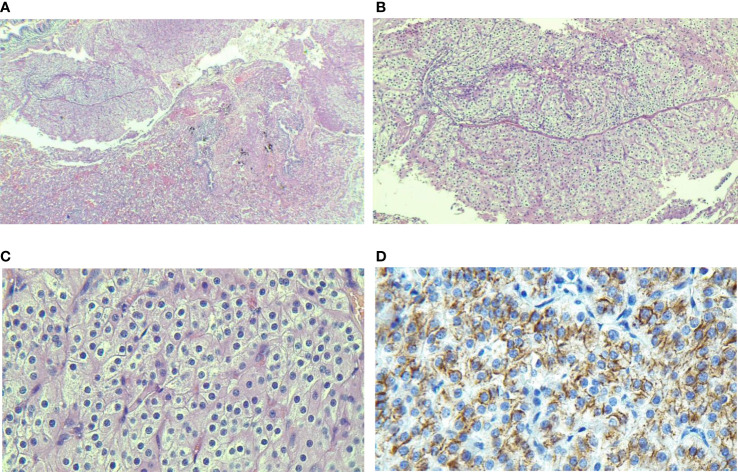
The pathological specimen confirms that parathyroid chief cells arrange in the adenoma tissues. **(A)** presence of well-defined nodule in lung parenchyma; **(B, C)** Monotonous population of cells with round nuclei, salt and pepper chromatin devoid of mitotic figures; **(D)** PTH cytoplasmic immunostaining.

Her laboratory exam in March 2021 showed a serum calcium level of 10.2 mg/dL, high serum iPTH, and normal Vitamin D level. The SPECT-CT scan showed nothing suggestive of parathyroid adenoma, neither in the neck nor in the mediastinum, but a solitary MIBI avid nodule was reported in the lower lobe of the right lung (11 x 5 mm) and was proposed to be ruled out for malignancy (**Figure 3A**). In fluorine-18 fluorodeoxyglucose positron emission tomography (18F-FDG PET), a faintly FDG-avid solid pulmonary nodule with a maximum standardized uptake value of 1.6 was noted in the right lower lobe. (**Figure 3B**). The patient was followed for three months by a pulmonologist, and a repeated CT Scan revealed a stable lesion. Then the patient was referred for surgery. Video-assisted thoracoscopic surgery (VATS) was done. Due to the absent nodule in 1st wedge resection by palpation, the surgical team did a right thoracotomy by modified axillary incision, and second wedge resection was taken after the nodule palpation was done just adjacent to the 1st wedge resection. She had a small gross nodule in the second wedge resection. The surgery was uneventful. The patient had postoperative hypocalcemia, which was managed by an appropriate dosage of calcium, and vitamin D. Pathological examination of the nodule which was 4 mm in the greatest dimension, confirmed the presence of parathyroid adenoma and a positive IHC staining for iPTH (**Figure 4**). Serological and symptomatic recovery was achieved, and the patient was discharged. Postoperatively hypoparathyroidism persisted, and she needed 2500 mg calcium carbonate and 1.25 mcg calcitriol per day. In other laboratory investigations, the patient’s creatinine was 0.9 mg/dl, and the CKD-EPI creatinine equation for glomerular filtration rate was 72.2 ml/min. Also, the 25(OH)D level was sometimes insufficient and almost in the normal range based on Endocrine Society criteria during follow-up. Written informed consent was obtained from the patient to publish this case report.

## Discussion

PHPT is associated with a number of long-term consequences, including decreased bone mineral density and an increased risk of fracture nephrolithiasis, kidney failure, and neuropsychiatric disorders such as weakness, easy fatiguability, drowsiness, and depression (5). PHPT is caused by a solitary parathyroid adenoma in around 80% of cases, and surgery is most often curative (5). Those who are not cured after surgery have persistent or recurrent PHPT. Persistent PHPT occurs when hypercalcemia does not resolve following parathyroidectomy, whereas recurrent PHPT occurs after a normocalcemic interval of at least six months following parathyroidectomy (6). Several factors have been associated with persistent PHPT, including an unexpert surgeon; the presence of ectopic or supernumerary glands; multiglandular disease; parathyroid carcinoma; and, rarely, parathyromatosis (6, 7).

Parathyromatosis is an uncommon but challenging cause of persistent PHPT; multiple nodules of benign hyperfunctioning parathyroid tissue are characterized as being spread throughout the neck and mediastinum. There are two possible explanations for the condition. The first hypothesis postulates that parathyromatosis occurs due to parathyroid tissue spillage and seeding inside the operative field during parathyroid surgery and accounts for most cases reported in the literature. The second hypothesis is that physiological stimuli induce hyperplasia of preexisting parathyroid rests of embryological origin. The diagnosis of parathyromatosis did not apply to our patient due to the location of the adenoma in the lungs and the pathological characteristics. Another factor contributing to parathyroid surgery failure is the presence of PHPT in multiple endocrine neoplasia (MEN) syndromes. PHPT associated with MEN syndrome is an asymmetric multiglandular, multifocal disorder; most cases are benign tumors represented by parathyroid hyperplasia or adenoma (8); it occurs equally in males and females that commonly affects individuals under 30 years of age (9). Before the final diagnosis, our patient underwent genetic testing, which ruled out the MEN syndromes.

Parathyroid adenoma is typically found in the neck region, particularly on the thyroid’s posterior capsule or in ectopic places (1). A prevalent and significant cause of persistent PHPT is an ectopic parathyroid gland, which occurs as a result of abnormal parathyroid migration during the early stages of fetal development (4). Superior parathyroid glands develop from the fourth branchial arch and migrate caudally with the thyroid gland. The inferior parathyroid glands arise from the same 3rd brachial arch as the thymus gland and accompany it on its descent, which explains their unusual location in the mediastinum. As a result of deviating from the normal pathway of descent, their ectopia can occur anywhere in the mediastinum up to the pericardium (4). To the best of our knowledge, no previous report has described the location of parathyroid adenoma outside of the embryonic descending pathway, which was observed in our patient’s lungs; she was evaluated after several surgeries of persistent PHPT following a MIBI avid nodule in her lung. Regarding the presence of a parathyroid adenoma in the lung, the patient was first suspected of having iPTH-secreting lung malignancies; the probability of malignancy was reduced due to the nodule’s stability during the follow-up and ruled out in the pathological examination. Finally, despite the distinct embryonic origins of lung and parathyroid tissue and the presence of adenoma reported outside the embryonic descent pathway, the histological pathology confirmed the diagnosis of parathyroid adenoma in lung tissue.

Diagnostic imaging should be conducted to localize the source of iPTH secretion. First-line localization procedures are neck ultrasonography, magnetic resonance imaging, computed tomography, and MIBI scan with or without SPECT/CT. The PET/CT is another imaging modality usually utilized in patients with persistent PHPT who are difficult to -localize (10). Two or more concordant studies should be obtained before undergoing surgery for persistent or recurrent PHPT. When this rule is followed, a success rate of up to 95% can be attained; however, there is no agreement on which two diagnostic modalities are the best in these conditions (5). The use of MIBI and SPECT alone or in combination with CT in sensitivity studies has been between 54 and 100 percent (3). The main pitfall in handling this patient after the first unsuccessful surgery was performing only one diagnostic modality for localizing the source of PTH secretion. Thus, for patients who have developed persistent PHPT, the patient’s prior medical history should be thoroughly evaluated and managed using a multidisciplinary approach by concordantly utilizing at least two diagnostic modalities.

There are two limitations in the presented case study. First, not using 11C-Choline or 18F-fluorocholine PET imaging, as recommended for examination of parathyroid tissue, due to lack of access. Second, there was no genetic examination of the hyperparathyroidism jaw tumor syndrome, which is one of the syndromes associated with primary hyperparathyroidism, even though the presented patient did not have other clinical manifestations of the syndrome, such as multiple ossifying fibromas of the maxilla and mandible, as well as renal and uterine tumors.

In conclusion, to our knowledge, this is the first case of persistent PHPT with parathyroid adenoma in the lung that has been documented in the literature. VATS was conducted safely and without complications, and blood calcium and iPTH levels immediately improved following the procedure. Aside from the conventional locations from the neck to the mediastinum, atypical regions should be assessed for persistent PHPT to avoid long-term complications and additional procedures.

## Data availability statement

The original contributions presented in the study are included in the article/supplementary material. Further inquiries can be directed to the corresponding author.

## Ethics statement

The studies involving human participants were reviewed and approved by Research Institute for Endocrine Sciences of Shahid Beheshti University of Medical Sciences. The patient provided their written informed consent to participate in this study.

## Author contributions

MV, AE, AA, and AZ were responsible for data collection and manuscript preparation. All authors approved this manuscript prior to submission. All authors contributed to the article and approved the submitted version.
